# Smartphone-based text obtained via passive sensing as it relates to direct suicide risk assessment

**DOI:** 10.1017/S0033291725001199

**Published:** 2025-05-09

**Authors:** Brooke A. Ammerman, Evan M. Kleiman, Connor O’Brien, Anne C. Knorr, Kerri-Anne Bell, Nilám Ram, Thomas N. Robinson, Bryon Reeves, Ross Jacobucci

**Affiliations:** 1 Department of Psychology, University of Wisconsin–Madison, Madison, WI, USA; 2 Department of Psychology, Rutgers University, Piscataway, NJ, USA; 3 Department of Psychology, University of Notre Dame, Notre Dame, IN, USA; 4 Departments of Psychology and Communications, Stanford University, Stanford, CA, USA; 5 Departments of Pediatrics, Medicine, and Epidemiology and Population Health, Stanford University, Stanford, CA, USA; 6 Department of Communications, Stanford University, Stanford, CA, USA; 7 Center for Healthy Minds, University of Wisconsin–Madison, Madison, WI, USA

**Keywords:** screenomics, passive sensing, digital phenotyping, intensive time sampling, suicidal thinking, suicidal planning

## Abstract

**Background:**

Recent research highlights the dynamics of suicide risk, resulting in a shift toward real-time methodologies, such as ecological momentary assessment (EMA), to improve suicide risk identification. However, EMA’s reliance on active self-reporting introduces challenges, including participant burden and reduced response rates during crises. This study explores the potential of Screenomics—a passive digital phenotyping method that captures intensive, real-time smartphone screenshots—to detect suicide risk through text-based analysis.

**Method:**

Seventy-nine participants with past-month suicidal ideation or behavior completed daily EMA prompts and provided smartphone data over 28 days, resulting in approximately 7.5 million screenshots. Text from screenshots was analyzed using a validated dictionary encompassing suicide-related and general risk language.

**Results:**

Results indicated significant associations between passive and active suicidal ideation and suicide planning with specific language patterns. Detection of words related to suicidal thoughts and general risk-related words strongly correlated with self-reported suicide risk, with distinct between- and within-person effects highlighting the dynamic nature of suicide risk factors.

**Conclusions:**

This study demonstrates the feasibility of leveraging smartphone text data for real-time suicide risk detection, offering a scalable, low-burden alternative to traditional methods. Findings suggest that dynamic, individualized monitoring via passive data collection could enhance suicide prevention efforts by enabling timely, tailored interventions. Future research should refine language models and explore diverse populations to extend the generalizability of this innovative approach.

## Introduction

Suicidal behaviors are associated with an economic burden upwards of $90 billion per year (Shepard et al., 2016), significant personal and emotional costs (Andriessen et al., 2017), and extensive resources dedicated to prevention efforts (National Institute of Health, 2018). In attempts to improve suicide risk identification, prediction, and intervention, researchers have shifted to identifying short windows of heightened suicide risk. Supporting this transition, technological advances have increased the accessibility of intensive time sampling methodologies, like ecological momentary assessment (EMA), aiding our understanding of suicide risk dynamics. Most notable is knowledge regarding the fluctuating nature of suicidal ideation (SI; Czyz, Horwitz, Arango, & King, 2019; Hallensleben et al., 2018; Kleiman et al., 2017), one of the top risk factors for future suicidal behavior. However, limitations of an active assessment approach to intensive time sampling (e.g., EMA; daily diary) have hindered reliable, real-time detection of suicide risk, a necessary step in momentary suicide risk intervention (i.e., Torous et al., 2018).

Sole reliance on active assessment methodologies to inform suicide risk identification presents several challenges. Foremost, this approach depends on individuals’ willingness and availability to provide self-reports of their experience(s) multiple times per day, resulting in a high burden that is not sustainable for extended periods (Ammerman & Law, 2022). Preliminary work also suggests that individuals may be less likely to respond to active assessments during times of suicidal crisis (Jacobucci, Ammerman, & Ram, 2024), potentially resulting in missed opportunities for risk detection and intervention. To balance such burden, many EMA studies restrict their temporal granularity, delivering EMA prompts every 3–4 hours (Ammerman & Law, 2022). Yet, the escalation of suicide risk can transpire in a matter of minutes (Deisenhammer et al., 2009; Millner, Lee, & Nock, 2017), requiring more temporally intensive, low-burden designs for risk identification and ultimate intervention.

Digital phenotyping is a promising avenue to improve real-time suicide risk detection (Onnela & Rauch, 2016). Digital phenotyping has seen an increase in application across clinical research broadly, with the term being previously applied to suicide risk profiles based on active assessments (i.e., EMA) (Kleiman et al., 2018). However, little research has specifically targeted passive assessment approaches to suicide risk detection; existing work has largely centered on passively collected psychophysiological responses (i.e., heart rate, electrodermal activity, daily steps; Czyz et al., 2023; Horwitz et al., 2022; Kleiman et al., 2018). An alternative approach is a novel form of digital phenotyping called Screenomics (Ram et al., 2020; Reeves et al., 2019; Reeves, Robinson, & Ram, 2020). Screenomics leverages the ubiquitous nature of smartphones by unobtrusively capturing screenshots from participants’ smartphones at an intensive rate (e.g., 5-second intervals during active phone use), resulting in comprehensive smartphone-based data streams (e.g., text extracted from screenshots) that does not require participant action. This low-burden, high-granularity data may demonstrate associations with actively assessed suicide risk.

While several elements may be extracted from screenshots (Ram et al., 2020; Reeves et al., 2019; Reeves et al., 2020), the use of text data has seen a recent rise in the field of suicide. Given high measurement reliability (Jodoin, 2003), the increasing prevalence of text-based communication, and the use of smartphones for numerous daily activities (i.e., reading, information gathering), text is a promising tool for suicidal risk detection. In prior research, various forms of social media postings (e.g., Facebook, Twitter) have been used to identify patterns in text (i.e., language content) to successfully classify those at high suicide risk (Bryan et al., 2018; Coppersmith, Leary, Crutchley, & Fine, 2018; Du et al., 2018; Guan et al., 2015). Downloaded text messages have also been used to retrospectively identify specific emotional content and stressors leading up to suicidal thoughts and behaviors (Glenn, Nobles, Barnes, & Teachman, 2020). Such prior work has underscored the potential of text-based communication in understanding suicide risk; however, to our knowledge, there has yet to be a comprehensive assessment of text-based information obtained from smartphones (i.e., across platforms; e.g., text messages, social media, web browsing), which may offer a more holistic view of suicide risk.

## Current study

The current study employs Screenomics – intensive, real-time data captured from participants’ smartphones, including text-based content across all platforms and applications that an individual uses – to explore a less intrusive, lower-burden method for assessing suicide risk. With growing interest in just-in-time interventions for suicide prevention (e.g., Coppersmith et al., 2022), which necessitate continuous, long-term monitoring, this study evaluates whether text data from smartphones may be related to self-reported suicide risk indicators, ultimately serving as a signal of suicide risk. Specifically, using a previously validated dictionary that includes words indicative of a crisis (Swaminathan et al., 2023), we aim to identify language patterns linked to real-time reports of passive and active SI, as well as suicidal planning. By introducing a scalable, innovative approach to suicide risk monitoring, this study has the potential to lay the groundwork for more timely and effective interventions.

## Method

### Participants

Participants were 79 individuals who reported past-month active SI or suicidal behaviors and owned an Android-based smartphone (required for the smartphone application). Participants were recruited via social media advertisements and community flyers from a mid-sized city in the Midwest. Participants ranged in age from 20–63 years (M = 35.15, SD = 11.07); 68.3% were female; 84.8% identified as white, 6.3% as Black, 6.3% as American Indian/Alaska Native, and 2.6% as another race; and 92.4% identified as non-Hispanic/Latino. Most participants (93.7%) had engaged in individual therapy or counseling in their lifetime. Regarding baseline diagnostic assessment of current clinical presentation, 83.8% of the sample met diagnostic criteria for at least one psychiatric disorder, and 64.9% met current diagnostic criteria for two or more disorders (M = 2.55, SD = 1.86). (Individuals with and without comorbid diagnoses were compared regarding the sum of dictionary-identified words across the course of the study. The sleep dictionary showed a significant difference between groups (t(53.08) = −2.72, p = .009), with comorbid participants (M = 73.49) exhibiting more than twice the sleep-related content compared to noncomorbid participants (M = 32.50). All other dictionaries showed no statistically significant differences between the two groups.) More specifically, 43.0% met diagnostic criteria for major depressive disorder, 34.2% for generalized anxiety disorder, 40.5% for panic disorder, 25.3% for social anxiety disorder, 32.9% for post-traumatic stress disorder, 22.8% for alcohol use disorder, and 25.3% for substance use disorder. Considering suicide risk history, 39.2% reported past week SI; 72.2% had a lifetime history of a suicide plan (38% in the past year, 15.1% in the past month); and 64.5% had a lifetime history of a suicide attempt (12.7% in the past year, 2.5% in the past month).

### Procedures

Participants completed an in-laboratory assessment consisting of a diagnostic interview (MINI; Sheehan et al., 1998), an interview of suicide risk history (i.e., Self-Injurious Thoughts and Behaviors Interview; Nock, Holmberg, Photos, & Michel, 2007), and an orientation to the out-of-lab procedures. The out-of-lab protocol included 28 days of EMA and screenshot capture. All participants who reported any SI via EMA during the out-of-lab protocol received an automated prompt with crisis resources; if participants reported elevated active SI (i.e., ≥ 4 on 5-point scale), a study team member reached out to conduct a comprehensive risk assessment. The authors assert that all procedures contributing to this work comply with the ethical standards of the relevant national and institutional committees on human experimentation and with the Helsinki Declaration of 1975, as revised in 2008. All study procedures were approved by the Institutional Review Board at the data collection site.

**Ecological Momentary Assessment.** Participants received six signal-contingent EMA prompts per day (i.e., randomized within 2-hour windows across a 12-hour block) via the LifeData app, each taking 3–4 minutes to complete. Each prompt had to be completed within 30 minutes. Participants received momentary compensation and were incentivized to complete 75% of daily surveys. Responses indicating moderate levels of SI prompted a reminder of crisis resources, whereas responses indicating potentially imminent risk triggered an email to the research team, resulting in a comprehensive suicide risk assessment conducted via phone. This sample had an overall EMA response rate of 68.8%.

**Screenshot Capture.** Screenshots were collected from participants’ smartphones via the *Screenlife Capture* application (Yee et al., 2023). Participants downloaded this application on their phones, which captures screenshots at 5-second intervals when the smartphone is in use, stores them on the local device, and then encrypts and securely transmits bundles of screenshots to research servers. Data were stored on secure, HIPPA-compliant servers internal to the first author’s institution. Participants deleted the *Screenlife Capture* application and all locally stored data at the end of the study period. The text data from each screenshot was then extracted for analysis (see Data Analysis section).

### Measures

**Suicidal ideation.** Four questions assessed momentary (i.e., ‘At this moment…’) passive SI (i.e., ‘Life is not worth living for me’; ‘There are more reasons to die than to live for me’) and active SI (i.e., ‘I think about taking my life’; ‘I want to die’), which have been previously validated (Forkmann et al., 2018). These items were answered on a 5-point Likert scale and summed to create passive SI and active SI composite scores.

**Suicidal planning.** Three questions assessed suicidal planning (i.e., ‘Considered a specific suicide method’; ‘Identified how to acquire your suicide method’; ‘Made other preparation for your death [e.g., wrote a suicide note, made arrangements]’) occurring over the EMA prompt interval (i.e., ‘Since the last prompt…’). These three items were adapted from the Beck Suicide Scale (Beck, Kovacs, & Weissman, 1979) and were answered on a 5-point Likert scale. All were summed to create a suicidal planning composite score.

### Data analysis

**Data Processing.** EasyOCR (Jaided, 2024), a deep learning-based optical character recognition system, was applied to each screenshot to extract text content. The Python implementation of EasyOCR uses a combination of text detection and recognition models to identify and transcribe text regions from images. During the manual inspection, we observed that text blocks with lower OCR confidence scores often contained inserted special characters rather than complete misreadings, making dictionary matching possible regardless of confidence thresholds. Therefore, we chose not to exclude any text blocks based on EasyOCR confidence scores.

Initial data analysis revealed that the mean smartphone use ‘session’ – which we defined as continuous use without breaks (i.e., no phone use) exceeding 30 seconds – lasted approximately 11 minutes (specifically, 696.73 seconds; 95% CI: 518.49, 874.96). Based on this, we analyzed screenshots in 10-minute intervals, combining dictionary counts for each 10-minute window before a given EMA assessment (extending back to the previous EMA prompt). For missed EMA prompts, we maintained the original time variables since these were used to model EMA response patterns (see below) while applying listwise deletion for missing outcomes in the remaining multilevel models. To maintain focus on proximal associations, we excluded rows where the time between screenshot collection and EMA responses exceeded 3 hours; this decision was made based on the average time between EMA prompts (i.e., 3.4 hours), resulting in primarily exclusion of screenshots leading up to a missed EMA prompt and those from the night/morning.

**Dictionary-Based Method.** A dictionary-based approach was selected over data-driven approaches to enhance the interpretability of findings. We applied a previously developed and validated dictionary, originally designed to support triaging crisis messages in real-time, based on chat data from a crisis line (Swaminathan et al., 2023). The system, which consists of 276 words, previously demonstrated high sensitivity (0.98) and positive predictive value (0.66), effectively distinguishing crisis-related from noncrisis content within telehealth settings (Swaminathan et al., 2023). To increase the specificity of our findings, two members of the study team with suicide expertise independently evaluated the complete dictionary and identified empirically and theoretically relevant themes; each word in the dictionary was assigned to one of the seven identified themes, which were used to generate subdictionaries. Any discrepancies in word placements within themes were discussed until a consensus was reached. This resulted in seven subdictionaries that were used in the current analyses: *suicidal thoughts* (41 words; e.g., ‘kill myself’; ‘no longer want to live’); *suicide methods* (75 words; e.g., ‘bridge’; ‘hang myself’); *alcohol and illicit substances* (26 words; e.g., ‘drinking’; ‘heroin’); *sleep* (8 words; e.g., ‘haven’t slept’; ‘nightmares’); *hopeless* (26 words; e.g., ‘defeated’; ‘no point’); *general risk* (93 words; e.g., ‘hate my life’; ‘depressed’); and *help-seeking* (8 words; e.g., ‘help me’; ‘want to be alive’). See Supplemental Table 1 for the full list of dictionary words.

Three complementary text-matching approaches were implemented to maximize detection accuracy: exact matching identified precise keyword occurrences (after lower-casing the OCR text), fuzzy matching (with a 95% similarity threshold) accounted for minor OCR errors and spelling variations, and lemmatized matching captured morphological variants of keywords. For each screenshot’s text content (at the OCR text block level), we calculated separate counts of keyword matches using each matching method across all dictionary categories. The analysis was implemented in Python, utilizing the NLTK library (Bird, Klein, & Loper, 2009) for text processing and lemmatization, and FuzzyWuzzy (Cohen & Schick, n.d.) for approximate string matching. Below, we only detail results for exact matching, as exact had very high correlations with lemmatization (mean = 0.98; range = 0.90–1.0), while fuzzy matching had correlations near zero with both other matching forms. The outcome of this approach is the word count for each dictionary, indicating the frequency with which words matching the dictionary criteria appear in the text within a given 10-minute interval.

**Data Analysis.** To examine the relationship between dictionary-detected language patterns and suicide risk, we calculated the mean rate of dictionary word usage in periods preceding EMAs. Dictionary rates were computed by summing the occurrences of words from each dictionary and normalizing by the time elapsed before each EMA. We compared these rates between responses of zero/nonzero for each suicide risk variable. Wilcoxon rank-sum tests were used to assess statistical significance between groups, given the nonnormal distribution of dictionary rates. To assess the strength and direction of relationships between dictionary-detected language patterns and suicide risk, we conducted Spearman rank correlations. Correlations were calculated between each dictionary’s usage rate and the three outcomes.

For each predictor, we disaggregated between- and within-person effects by calculating: (1) each participant’s mean proportion of 10-minute intervals containing any dictionary words (termed *between-person* effects) and (2) the interval-specific deviations from this individual average (termed *within-person* effects). We opted for dichotomizing dictionary count for both the between- and within-person specifications, given the extreme zero inflation of each 10-minute window dictionary count. We tested models that included multiple within-person specifications (namely, included deviations in count from person-specific means); however, there was a high correlation between these variables given the low base rate of nonzero counts. We found improved model convergence with the dichotomous relative to continuous coding. Relationships within the data were examined using generalized multilevel models that accommodated the nested nature of the data (repeated assessments nested within persons) and the nonnormal distributions of the outcome variables, which were characterized by a high number of zero values (Moghimbeigi, Eshraghian, Mohammad, & Mcardle, 2008; Snijders & Bosker, 2011). Given this, we specified zero-inflated negative binomial (ZINB; zero-inflated Poisson models had a high rate of convergence problems) distributions for each of the suicide risk outcomes. These models consisted of two parts (zero versus nonzero part and the count part; referred to as excess zero and count in-text, respectively). The resulting model part estimates warrant different interpretations: estimates from the nonzero (‘excess zero’ in text) part explain the probability that an observation falls into the category of excess zeros (logistic model); estimates from the count part of the model (negative binomial model; ‘count’ in text) describe the frequency of nonzero outcomes.

This model took the general form:

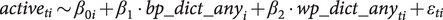




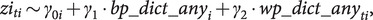

where for each dictionary (*dict*), both the within- ( 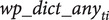 ) and between-person ( 
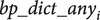
 ) variables are specified for both parts of the outcome (Count: 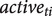 ; 0/1 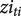 ).

To clarify the interpretation of model outputs, we provide a hypothetical example using the *hopeless* dictionary and the outcome of passive suicidal ideation. Zero-inflated negative binomial (ZINB) models consist of two components: the zero-inflation component and the count component. Each helps us understand different aspects of the relationship between predictors and outcomes. The zero-inflation component addresses the likelihood of excess zeros. In our context, an estimate from this portion represents the relationship between dictionary use and the probability that a participant reports *no* passive suicidal ideation. For example, a negative coefficient for the *within-person hopeless* dictionary variable in the zero-inflation component would indicate that on days when an individual uses more hopeless language than their personal average, they are less likely to report zero passive suicidal ideation—in other words, they have a higher probability of experiencing at least some passive suicidal ideation. It’s important to note that unlike common practice in logistic regression, the reference case here is ‘zero’ (absence of the outcome), so coefficient interpretations may appear counterintuitive. Conversely, the count component models the intensity of the outcome when it is present. An estimate from this portion reflects the relationship between dictionary use and the severity of passive suicidal ideation on days when such ideation occurs. For instance, a positive coefficient for the *between-person hopeless* dictionary variable in the count component would suggest that individuals who generally exhibit more hopeless language throughout the study period tend to report higher levels of passive suicidal ideation when experiencing any such ideation. This dual-component approach allows us to distinguish between: (1) factors influencing whether any suicide risk is present, and (2) factors affecting the severity of that risk when it occurs – providing a more nuanced understanding of suicide risk factors than traditional regression approaches.

The full set of generalized multilevel models was estimated in a Bayesian framework using the *brms* package (Bürkner, 2017) in R (R Core Team, 2021). Model convergence was assessed using the *Rhat* metric and effective sample size (ESS; Vehtari, Gelman, & Gabry, 2017). To achieve an adequate number of samples for each parameter, 10 chains were used, with at least 200 samples per chain (100 burn-in). Additionally, we tested combined models that included all dictionary counts in one model. However, we opted against this specification due to the correlation across some dictionary counts and convergence issues.

## Results

### Preliminary analysis

The average screenshot image count per participant was 92,613 (median = 85,795; range = 2,185–431,623). Regarding each dictionary, the suicide methods dictionary had the highest percentage of nonzero entries within the 10-minute intervals across the study (45.68%), followed by the general risk dictionary (19.83%), suicidal thoughts dictionary (9.07%), alcohol and illicit substances dictionary (5.64%), help-seeking dictionary (1.09%), hopeless dictionary (0.36%), and the sleep dictionary (0.33%). Correlations between screenshot image count and word count per subdictionary were small in magnitude, with the strongest correlation being with suicide methods (r = .24), followed by general risk (r = .15) and suicidal thoughts (r = .11), with all other correlations r < .10. Nonzero passive SI was reported on 35.0% of EMA prompts; nonzero active SI on 32.72% of prompts; and nonzero suicidal planning on 13.11% of prompts.

Analysis of dictionary rates revealed significant differences between high (i.e., > threshold) and low (i.e., ≤ threshold) suicide risk across multiple dictionaries. For both passive and active SI, participants reporting higher SI showed significantly elevated rates of usage from the general risk, suicide methods, alcohol and illicit substances, and suicidal thoughts dictionaries. No differences were found for suicidal planning. See Table 1. Similarly, spearman correlations revealed small but significant associations for passive and active SI with the general risk, suicide methods, alcohol and illicit substances, and suicidal thoughts dictionaries. There was also a correlation between the help-seeking dictionary and passive SI; no significant correlations were found for suicidal planning. See Table 2.

**Table 1.** Mean dictionary rates by suicide risk outcome

Dictionary	Passive SI (Low)	Passive SI (High)	P-Value	Active SI (Low)	Active SI (High)	P-Value	SP (Low)	SP (High)	P-Value
General risk	0.98	1.17	< .001***	0.97	1.21	< .001***	1.02	1.22	0.30
Help-seeking	0.03	0.06	0.13	0.04	0.06	0.49	0.04	0.05	0.37
Hopeless	0.01	0.03	0.18	0.01	0.04	0.14	0.02	0.05	0.39
Suicide methods	3.42	3.70	< .001***	3.42	3.70	< .001***	3.58	3.09	0.12
Sleep	0.02	0.01	0.53	0.02	0.01	0.88	0.02	0.02	0.89
Alcohol and Substance	0.22	0.25	< .001***	0.21	0.26	< .001***	0.23	0.22	0.28
Suicidal thoughts	0.37	0.54	< .001***	0.36	0.57	< .001***	0.43	0.50	0.30

*Note*: SI, suicidal ideation; SP, suicidal planning; Low, mean rate when outcome ≤ threshold; High, mean rate when outcome > threshold; *=p < .05. **=p < .01. ***=p < .001.

**Table 2.** Spearman correlation between dictionary usage and suicide risk outcomes

Dictionary	1.	2.	3.	4.	5.	6.	Passive SI	Active SI	SP
1. General risk							0.05*	0.07*	0.02
2. Help-seeking	.06						0.03*	0.01	0.01
3. Hopeless	.03	.004					0.02	0.01	0.01
4. Suicide methods	.21	.04	.03				0.05*	0.05*	−0.01
5. Sleep	.04	.01	.002	.03			−0.01	0.00	−0.00
6. Alcohol and substance	.14	.04	.01	.14	.01		0.05*	0.05*	0.01
7. Suicidal thoughts	.13	.04	.02	.18	.02	.08	0.06*	0.07*	0.01

*Note*: SI, suicidal ideation; SP, suicidal planning; *p < .05.

### Passive SI

There was a significant, negative, excess zero, between-person effect for the hopeless dictionary in predicting passive SI (Supplemental Table 3). There was a significant, positive, count, within-person effect, and a significant, positive, excess zero, within-person effect for the general risk dictionary predicting passive SI (Supplemental Table 5). There was a significant, positive, count, between-person effect and a significant, negative, excess zero, between-person effect for the suicidal thoughts dictionary predicting passive SI (Supplemental Table 8). There were no significant effects for help-seeking (Supplemental Table 2), suicide methods (Supplemental Table 4), sleep (Supplemental Table 6), or alcohol or illicit substances (Supplemental Table 7) dictionaries.

### Active SI

There was a significant, positive, excess zero, within-person effect for the suicide methods dictionary predicting active SI (Supplemental Table 11). There was a significant, positive, count, within-person effect, and a significant, negative, excess zero, between-person effect for the general risk dictionary predicting active SI (Supplemental Table 12). There was a significant, negative, excess zero, between-person effect for the suicidal thoughts dictionary predicting active SI (Supplemental Table 15). There were no significant effects for hopelessness (Supplemental Table 10), sleep (Supplemental Table 13), alcohol and illicit substances (Supplemental Table 14), or help-seeking (Supplemental Table 9) dictionaries.

### Suicidal planning

There was a significant, negative, excess zero, within-person effect for the general risk dictionary predicting suicidal planning (Supplemental Table 19). There was a significant, positive, count, within-person effect for the sleep dictionary predicting suicidal planning (Supplemental Table 20). There was a significant, negative, excess zero, within-person effect for the suicidal thoughts dictionary predicting suicidal planning (Supplemental Table 22). There were no significant effects for help-seeking (Supplemental Table 16), hopelessness (Supplemental Table 17), suicide methods (Supplemental Table 18), or alcohol and illicit substances (Supplemental Table 21) dictionaries.

## Discussion

This study evaluated whether language patterns extracted from smartphone-based text were associated with self-reported passive SI, active SI, and suicidal planning. Findings demonstrate the potential promise of dictionary-based approaches for real-time suicide risk detection, with consistent associations observed for suicidal thoughts and general risk dictionaries, and outcome-specific effects identified in other subdictionaries. These results align with prior research on text-based communication for suicide risk detection (e.g., Bryan et al., 2018; Glenn et al., 2020) and advance this work by showing the feasibility of leveraging diverse text sources across platforms in real time. Additionally, the findings provide initial support for the potential of smartphone-based text as a low-burden, scalable complement to traditional EMA methods for suicide risk assessment.

The suicidal thoughts dictionary was consistently linked to suicide risk outcomes. Greater detection of suicidal thought words was associated with more severe passive SI and higher likelihood of active SI and suicidal planning. Passive and active SI effects were between person, indicating individuals with higher average detection reported more passive and active SI. In contrast, suicidal planning reflected within-person effects, with spikes in word detection linked to planning behaviors. These findings highlight the potential value of smartphone-based suicide-related content, as well as the benefits of examining both between- and within-person variance. Screenomics captures cross-platform text, including direct and indirect engagement with suicide-related content, enabling more comprehensive detection of suicide risk. For example, in addition to capturing direct conversations regarding suicide risk (i.e., via text messages and social media), it also captures engagement with online support platforms (i.e., IMAlive, 7 Cups of Tea, r/SuicideWatch; e.g., Lewis & Seko, 2016; Memon, Sharma, Mohite, & Jain, 2018), as well as other informative smartphone use behavior (i.e., online searches for suicide information; Biddle et al., 2012; Choi, Han, & Hong, 2023), which may ultimately open doors for supportive interventions.

The general risk factors dictionary was linked to all three suicide risk outcomes. Within-person effects showed that spikes in general risk word detection were associated with passive SI, active SI, and suicidal planning, while a between-person effect indicated individuals with higher overall detection reported more passive SI. These findings align with prior research on the difficulty of predicting suicide risk using single constructs (Franklin et al., 2017). By combining diverse risk indicators (e.g., ‘no energy’, ‘depressed’, ‘lonely’; e.g., Cai et al., 2021; Shoib et al., 2023; Schafer et al., 2022), the general risk factors dictionary addresses this challenge – capturing both specific and broad expressions of distress that may signal suicide risk. Unexpectedly, within-person increases in general risk words were linked to lower odds of reporting passive SI. Elevated general risk language may reflect increased cognitive engagement with distress (e.g., venting or catastrophizing), temporarily mitigating passive SI; however, the consistent positive relationships with active SI and suicidal planning, along with research linking venting to negative mental health outcomes (Marr, Zainal, & Newman, 2022;), make this explanation less likely. It is possible that passive SI is a more dynamic process (e.g., more fleeting thoughts), such that the timing of our EMA prompts may not have captured the complete risk picture, limiting the interpretation of our findings. The broad emotional language included in the general risk dictionary may partly explain these findings. For example, some words (e.g., ‘depressed’, ‘lonely’) might align with high-severity passive SI but fall short of predicting its presence, while others (e.g., ‘hate my life’) may reflect intense distress linked to active SI or planning. These results may also highlight variability in how individuals experience and express passive SI (Czyz et al., 2022; Kleiman et al., 2018). To better understand these patterns, future research should explore which specific words in the dictionary drive these divergent relationships and how general risk language evolves with passive SI severity and transitions between passive and active SI over time.

Several outcome-specific findings emerged. Between-person analyses showed that higher overall detection of hopelessness-related words predicted passive SI. These findings offer information that may potentially expand our understanding of the constructs central to the Interpersonal Theory of Suicide (Van Orden et al., 2010), which links hopelessness to active SI through thwarted belongingness and perceived burdensomeness. In contrast, the hopelessness dictionary captures broader despair (e.g., ‘given up’, ‘can’t take it’, ‘defeated’), possibly helping to explain the link to passive SI. This finding is supported by a meta-analysis suggesting that general hopelessness is more strongly associated with passive than active SI (Liu, Bettis, & Burke, 2020); together, this may highlight the role of hopelessness across types of SI (i.e., passive, active). It is also important to consider the intensive data structure underlying the current findings, uncovering dynamic associations not often accounted for in theoretical models. As prior intensive sampling research has shown a robust link between hopelessness and passive SI (Hallensleben et al., 2019), the current study builds on these findings, demonstrating that passively collected text data may be an alternative way to capture hopelessness at an intensive time scale, reducing self-report burdens and providing a scalable tool for early detection and intervention.

Higher-than-usual detection of sleep-related words (e.g., ‘haven’t slept’, ‘nightmares’) was associated with greater severity of suicidal planning. While research has linked sleep disorders to SI and suicide attempts (Drapeau & Nadorff, 2017), meta-analyses show that general sleep problems (e.g., difficulty falling asleep, staying asleep) are strongly associated with suicidal planning (OR = 2.6; Wojnar et al., 2009). These findings align with longitudinal studies linking sleep disturbances, duration, and quality to next-day SI (Brüdern et al., 2022; Kivelä, Van der Does, & Antypa, 2024; Littlewood et al., 2019). Given biases in self-reported sleep data, particularly among individuals with mood disorders (Biddle et al., 2015; Lauderdale et al., 2008), text-based detection of sleep-related language may offer a potential objective tool for assessing sleep’s role in suicidal planning risk.

The findings for the suicide methods dictionary were unexpected, as greater detection of these words was associated with the absence of active SI, contrary to expectations. One possibility is that discussing suicide methods may serve as a coping mechanism (e.g., venting or cognitive diffusion), reducing SI. While venting has been linked to increased suicide risk in some studies (Baer et al., 2022), other research suggests that talking with peers, providers, or crisis lines reduces daily suicidal urges (Al-Dajani, Horwitz, & Czyz, 2022). Alternatively, these terms may often reflect nonsuicidal content. Words like ‘bridge’ may appear in unrelated contexts, or phrases like ‘hang me’ could be sarcastic. Future research should examine specific terms within the dictionary to better understand these patterns and their potential meanings.

This study has several strengths, including the use of temporally granular data and text integration across various smartphone activities, offering a comprehensive view of suicide risk. However, limitations should be noted. The sample, drawn from a Midwest city, was homogenous, highly engaged in treatment, and reported high psychopathology comorbidity, limiting generalizability to more diverse populations, including those of lower clinical severity or with recent onset of suicidal ideation. Excluding iOS users, who differ demographically from Android users (e.g., higher income, younger age; Howarth, 2024), further impacts generalizability. Dictionary-based methods, while interpretable, rely on predefined word lists, limiting flexibility and the ability to assess causal relationships (e.g., exposure to suicide-related content increasing suicidal thoughts; Marchant et al., 2021). Further, while the combined dictionary has previously been validated, the current analysis implemented newly subsetted subdictionaries to enhance finding interpretation. Future work could complement this approach with more flexible natural language processing techniques, such as linguistic feature extraction or machine learning models, which have seen a recent increase in application in suicide research (i.e., Arowosegbe & Oyelade, 2023). Similarly, integrating other passive smartphone data sources (e.g., geolocation, activity levels, circadian patterns) may enrich the understanding of how digital behaviors relate to suicide risk. The analyses also did not account for co-occurrence of all three suicide-related outcomes, leaving the relationship between subdictionaries and comorbid ideation and planning unclear. Optional screenshot disabling may have introduced self-selection bias, potentially underestimating associations between text data and suicide risk. Additionally, the source of text (e.g., messaging apps, social media) was not differentiated, preventing platform-specific insights. Finally, reliance on OCR likely overlooked nuanced indicators of risk conveyed through images, emojis, or other nontextual content.

This study highlights the potential of smartphone-based text data to enhance real-time suicide risk detection, offering a scalable, low-burden approach that may, ultimately, be integrated into clinical care and crisis response systems. The differential predictive effects across dictionaries, such as general risk and suicidal thoughts, suggest that specific language patterns may align with distinct components of SI and suicidal planning, which, after replication, has the potential to pave the way for tailored interventions in the future. Moreover, the ability to detect within-person deviations in risk-related language emphasizes the promise of individualized, dynamic monitoring, moving beyond traditional static assessments. This initial study lays the foundation of a burgeoning research area suggesting that Screenomics may be a valid and feasible avenue for real-time data collection that could facilitate immediate support through crisis hotlines, instant messaging services, or connection with personal contacts, while also enabling the development of personalized treatment strategies. However, given the preliminary nature of the current results, future research is needed to explore the generalizability of findings. Despite these challenges, this study represents a critical step forward in leveraging digital technology for suicide prevention, offering innovative methods to address a significant public health challenge.

## Supporting information

Ammerman et al. supplementary material Ammerman et al. supplementary material

## Supplementary material

The supplementary material for this article can be found at http://doi.org/10.1017/S0033291725001199.
