# Supplementary material for: Smartphone-based text obtained via passive sensing as it relates to direct suicide risk assessment
Source: Psychol Med. 2025 May 9;55:e144. doi: 10.1017/S0033291725001199 (PMC12094652; doi:10.1017/S0033291725001199)
Supplement: Ammerman et al. supplementary material [file S0033291725001199sup001.docx]

Supplemental Table 1. Complete List of Sub-Dictionary Words

| **Suicidal Thoughts** | **Suicide Methods** | **Alcohol and Illicit Alcohol & Illicit Substances** | **Sleep** | **Help-Seeking** | **Hopeless** | **General Risk** |
| --- | --- | --- | --- | --- | --- | --- |
| Suicide | Excedrin | Relapse | exhausted | Help me | Helpless | Crisis |
| Suicidal | 800 mg | Relapsed | haven't slept | Emergency | Give up | Feel terrible |
| Unsafe | Bathtub | inject myself | insomnia | hospital | Stop the pain | Worst day of my life |
| Hurt myself | Ibuprofens | drink the whole bottle | any sleep | lifeline | Can't this anymore | Midnight |
| Harm myself | Electrocute | drunk | sleep deprivation | national hotline | 13th reason | 11:11 |
| Kill | Electrocution | drinking | can't wake up | I need counseling | really done | Nightstand |
| Kill myself | Bridge | hangover | nightmare | I need help | given up | Vampire |
| Die | Railroad | drink myself |  | want to be alive | can’t take it | Worthless |
| Death | Acetaminophen | abuse |  |  | no more hope | Torture |
| Don't want to be here | cut myself | addiction |  |  | no point | Failure |
| End it all | Slit | addicted |  |  | no hope | Hate my life |
| End my life | Hit by a bus | booze |  |  | Hopeless | Kurt Cobain |
| Hadn't been born | Drown | tablet |  |  | lack of hope | fucking |
| Sleep forever | Rope | alcohol |  |  | hopelessness | freaking |
| My time has come | stab my throat | narcotics |  |  | defeated | can't even |
| No longer want to live | slash my throat | vodka |  |  | can't go on | ugh |
| commit suicide | cut my throat | drugs |  |  | can't take it anymore | crap |
| suicidal thoughts | slash my neck | drug |  |  | fight is over | pissed |
| suicidal urges | cut my forearm | pain killer |  |  | fight this anymore | angry |
| I'm going to sleep forever | slash my forearm | pain killers |  |  | I'm done | wtf |
| leave everything behind | slit my forearm | cocaine |  |  | I’m just done | shit |
| I just want this all to end | pistol | heroin |  |  | So done | I hate my |
| meet the reaper | Syringe | whiskey |  |  | end tonight | tears |
| No reason to live | Antifreeze | lsd |  |  | ends tonight | stop crying |
| Never wake up | OD | meth |  |  | need a break | cry myself |
| Nothing left to live for | Overdose | shrooms |  |  | give in | into a coma |
| attempt | Train |  |  |  |  | get out of bed |
| dead | Tablets |  |  |  |  | no energy |
| off myself | Hang |  |  |  |  | low energy |
| suicid | Noose |  |  |  |  | can't get up |
| go to sleep forever | Revolver |  |  |  |  | this urge |
| better off dead | 800mg |  |  |  |  | this is the only way |
| tired of life | Shoot |  |  |  |  | nobody can stop me |
| can't go on living like this | Stab |  |  |  |  | you can't stop me |
| not worth living | going to cut |  |  |  |  | never been this close |
| suicide pact | start cut |  |  |  |  | never been this bad |
| take my life | wanna cut |  |  |  |  | Feeling trapped |
| take my last breath | Urge to cut |  |  |  |  | Lost everything |
| end everything | hang myself |  |  |  |  | going downhill |
| end it | walk into traffic |  |  |  |  | gone downhill |
| go into the great unknown | step into traffic |  |  |  |  | resist |
|  | Shotgun |  |  |  |  | temptation |
|  | Weapon |  |  |  |  | finish it |
|  | gas myself |  |  |  |  | grave |
|  | poison myself |  |  |  |  | passed away |
|  | drink bleach |  |  |  |  | heaven |
|  | Bleach |  |  |  |  | lost my |
|  | cut my wrists |  |  |  |  | lost a |
|  | slash my wrists |  |  |  |  | depressed |
|  | slit my wrists |  |  |  |  | cry |
|  | take all these pills |  |  |  |  | depression |
|  | take a handfull of these pills |  |  |  |  | fucked |
|  | swallow these pills |  |  |  |  | bullshit |
|  | at my throat |  |  |  |  | lose my mind |
|  | pull the trigger |  |  |  |  | disappoint |
|  | sharp |  |  |  |  | Fail |
|  | rifle |  |  |  |  | lonely |
|  | ar-15 |  |  |  |  | loneliness |
|  | magnum |  |  |  |  | one care |
|  | firearm |  |  |  |  | wants me to die |
|  | suicide plan |  |  |  |  | wants me to be dead |
|  | jump off |  |  |  |  | struggle |
|  | suicide note |  |  |  |  | struggling |
|  | suicide letter |  |  |  |  | not living |
|  | bleed out |  |  |  |  | negative thoughts |
|  | gun |  |  |  |  | threat |
|  | knife |  |  |  |  | threatened |
|  | broken glass |  |  |  |  | It's too late |
|  | blade |  |  |  |  | so alone |
|  | razorblade |  |  |  |  | the worst it's ever been |
|  | razor |  |  |  |  | in my hands |
|  | blow my brains out |  |  |  |  | it's too much |
|  | suffocate |  |  |  |  | with life |
|  | burn |  |  |  |  | self-harm |
|  | start burn |  |  |  |  | self harm |
|  |  |  |  |  |  | self-injury |
|  |  |  |  |  |  | self injury |
|  |  |  |  |  |  | despair |
|  |  |  |  |  |  | unhappy |
|  |  |  |  |  |  | freaking out |
|  |  |  |  |  |  | terrified |
|  |  |  |  |  |  | can’t breathe |
|  |  |  |  |  |  | freak out |
|  |  |  |  |  |  | I don't want to |
|  |  |  |  |  |  | falling apart |
|  |  |  |  |  |  | losing it |
|  |  |  |  |  |  | no one cares |
|  |  |  |  |  |  | I have no one |
|  |  |  |  |  |  | the end |
|  |  |  |  |  |  | weep |
|  |  |  |  |  |  | sadness |
|  |  |  |  |  |  | lost all joy |
|  |  |  |  |  |  | Hate |

Supplemental Table 2. Help-Seeking Dictionary Predicting Passive Suicidal Ideation

| Parameter | Estimate | SE | 95% CI Lower | 95% CI Upper | Bulk ESS | Rhat |
| --- | --- | --- | --- | --- | --- | --- |
| Count, Random Intercept | 0.922 | 0.127 | 0.715 | 1.197 | 403 | 1.022 |
| ZI, Random Intercept | 4.256 | 0.512 | 3.382 | 5.393 | 402 | 1.022 |
| Count, Intercept | 0.405 | 0.135 | 0.127 | 0.648 | 886 | 1.005 |
| ZI, Intercept | 0.188 | 0.494 | -0.837 | 1.118 | 732 | 1.016 |
| Count, Help Seeking, Between-Person | 0.245 | 0.132 | -0.010 | 0.508 | 950 | 1.006 |
| Count, Help Seeking, Within-Person | -0.042 | 0.049 | -0.148 | 0.047 | 989 | 1.013 |
| ZI, Help Seeking, Between-Person | -0.454 | 0.552 | -1.508 | 0.624 | 796 | 1.008 |
| ZI, Help Seeking. Within-Person | -0.089 | 0.191 | -0.445 | 0.276 | 922 | 1.009 |
| Shape | 533.578 | 157.044 | 283.856 | 897.219 | 1119 | 1.015 |

*Note:* ZI = zero-inflated; SE = Standard Error, CI = Confidence Interval; ESS = Effective Sample Size; Shape is a dispersion parameter

Supplemental Table 3. Hopelessness Dictionary Predicting Passive Suicidal Ideation

| Parameter | Estimate | SE | 95% CI Lower | 95% CI Upper | Bulk ESS | Rhat |
| --- | --- | --- | --- | --- | --- | --- |
| Count, Random Intercept | 0.948 | 0.127 | 0.740 | 1.258 | 423 | 1.027 |
| ZI, Random Intercept | 4.105 | 0.511 | 3.280 | 5.294 | 493 | 1.005 |
| Count, Intercept | 0.401 | 0.132 | 0.130 | 0.636 | 902 | 1.000 |
| ZI, Intercept | 0.119 | 0.495 | -0.793 | 1.057 | 875 | 1.019 |
| Count, Hopeless, Between-Person | 0.043 | 0.112 | -0.173 | 0.269 | 794 | 1.025 |
| Hopeless, Within-Person | 0.024 | 0.082 | -0.131 | 0.181 | 1215 | 1.015 |
| Zero Inflation Hopeless, Between-Person | -2.025 | 0.850 | -3.909 | -0.669 | 536 | 1.007 |
| Zero Inflation Hopeless, Within-Person | 0.390 | 0.353 | -0.322 | 1.036 | 1718 | 1.019 |
| Shape | 529.592 | 160.265 | 295.413 | 893.094 | 1313 | 1.010 |

*Note:* ZI = zero-inflated; SE = Standard Error, CI = Confidence Interval; ESS = Effective Sample Size; Shape is a dispersion parameter

Supplemental Table 4. Suicide Methods Dictionary Predicting Passive Suicidal Ideation

| Parameter | Estimate | SE | 95% CI Lower | 95% CI Upper | Bulk ESS | Rhat |
| --- | --- | --- | --- | --- | --- | --- |
| Count, Random Intercept | 0.955 | 0.138 | 0.732 | 1.273 | 301 | 1.030 |
| ZI, Random Intercept | 4.279 | 0.506 | 3.419 | 5.328 | 508 | 1.015 |
| Count, Intercept | 0.394 | 0.138 | 0.108 | 0.652 | 627 | 1.010 |
| ZI, Intercept | 0.102 | 0.519 | -0.933 | 1.080 | 895 | 1.014 |
| Count, Suicide Method, Between-Person | 0.129 | 0.133 | -0.134 | 0.385 | 690 | 1.013 |
| Count, Suicide Method, Within-Person | 0.007 | 0.012 | -0.016 | 0.031 | 1059 | 1.021 |
| ZI, Suicide Method, Between-Person | 0.359 | 0.529 | -0.677 | 1.384 | 747 | 1.006 |
| ZI, Suicide Method, Within-Person | 0.066 | 0.041 | -0.015 | 0.151 | 1070 | 1.012 |
| Shape | 535.874 | 166.928 | 284.606 | 921.728 | 1460 | 1.013 |

*Note:* ZI = zero-inflated; SE = Standard Error, CI = Confidence Interval; ESS = Effective Sample Size; Shape is a dispersion parameter

Supplemental Table 5. General Risk Dictionary Predicting Passive Suicidal Ideation

| Parameter | Estimate | SE | 95% CI Lower | 95% CI Upper | Bulk ESS | Rhat |
| --- | --- | --- | --- | --- | --- | --- |
| Count, Random Intercept | 0.948 | 0.176 | 0.733 | 1.295 | 253 | 1.030 |
| ZI, Random Intercept | 4.284 | 0.550 | 3.364 | 5.367 | 407 | 1.013 |
| Count, Intercept | 0.385 | 0.184 | 0.069 | 0.643 | 506 | 1.019 |
| ZI, Intercept | 0.116 | 0.518 | -0.987 | 1.113 | 503 | 1.028 |
| Count, General Risk, Between-Person | 0.117 | 0.118 | -0.107 | 0.355 | 829 | 1.005 |
| Count, General Risk, Within-Person | 0.054 | 0.027 | 0.026 | 0.086 | 809 | 1.014 |
| ZI, General Risk, Between-Person | -0.826 | 0.538 | -1.863 | 0.228 | 807 | 1.017 |
| ZI, General Risk, Within-Person | 0.089 | 0.516 | 0.019 | 0.229 | 792 | 1.013 |
| shape | 546.756 | 188.672 | 276.267 | 937.353 | 957 | 1.015 |

*Note:* ZI = zero-inflated; SE = Standard Error, CI = Confidence Interval; ESS = Effective Sample Size; Shape is a dispersion parameter

Supplemental Table 6. Sleep Dictionary Predicting Passive Suicidal Ideation

| Parameter | Estimate | SE | 95% CI Lower | 95% CI Upper | Bulk ESS | Rhat |
| --- | --- | --- | --- | --- | --- | --- |
| Count, Random Intercept | 0.964 | 0.135 | 0.742 | 1.277 | 384 | 1.008 |
| ZI, Random Intercept | 4.316 | 0.495 | 3.429 | 5.401 | 450 | 1.021 |
| Count, Intercept | 0.399 | 0.136 | 0.118 | 0.662 | 636 | 1.013 |
| ZI, Intercept | 0.146 | 0.488 | -0.869 | 1.047 | 868 | 1.017 |
| Count, Sleep, Between-Person | 0.046 | 0.120 | -0.184 | 0.279 | 957 | 1.007 |
| Count, Sleep, Within-Person | -0.144 | 0.101 | -0.350 | 0.039 | 910 | 1.004 |
| ZI, Sleep, Between-Person | -0.183 | 0.536 | -1.231 | 0.852 | 865 | 1.003 |
| ZI, Sleep, Within-Person | -0.233 | 0.358 | -0.966 | 0.449 | 1752 | 1.025 |
| Shape | 538.016 | 166.247 | 286.662 | 923.726 | 1047 | 1.013 |

*Note:* ZI = zero-inflated; SE = Standard Error, CI = Confidence Interval; ESS = Effective Sample Size; Shape is a dispersion parameter

Supplemental Table 7. Alcohol and Illicit Substances Dictionary Predicting Passive Suicidal Ideation

| Parameter | Estimate | SE | 95% CI Lower | 95% CI Upper | Bulk ESS | Rhat |
| --- | --- | --- | --- | --- | --- | --- |
| Count, Random Intercept | 0.956 | 0.138 | 0.729 | 1.283 | 380 | 1.021 |
| ZI, Random Intercept | 4.293 | 0.519 | 3.437 | 5.444 | 601 | 1.010 |
| Count, Intercept | 0.412 | 0.135 | 0.128 | 0.655 | 889 | 1.009 |
| ZI, Intercept | 0.146 | 0.506 | -0.864 | 1.110 | 834 | 1.018 |
| Count, Alcohol & Illicit Substance, Between-Person | -0.040 | 0.127 | -0.293 | 0.190 | 905 | 1.014 |
| Count, Alcohol & Illicit Substance, Within-Person | 0.016 | 0.025 | -0.033 | 0.066 | 1140 | 0.999 |
| ZI, Alcohol & Illicit Substance, Between-Person | -0.658 | 0.566 | -1.821 | 0.421 | 729 | 1.009 |
| ZI, Alcohol & Illicit Substance, Within-Person | 0.040 | 0.092 | -0.142 | 0.214 | 954 | 1.011 |
| Shape | 538.603 | 160.933 | 295.085 | 899.269 | 1361 | 1.005 |

*Note:* ZI = zero-inflated; SE = Standard Error, CI = Confidence Interval; ESS = Effective Sample Size; Shape is a dispersion parameter

Supplemental Table 8. Suicidal Thoughts Dictionary Predicting Passive Suicidal Ideation

| Parameter | Estimate | SE | 95% CI Lower | 95% CI Upper | Bulk ESS | Rhat |
| --- | --- | --- | --- | --- | --- | --- |
| Count, Random Intercept | 0.895 | 0.123 | 0.684 | 1.161 | 354 | 1.020 |
| ZI, Random Intercept | 4.045 | 0.466 | 3.251 | 5.068 | 379 | 1.020 |
| Count, Intercept | 0.393 | 0.126 | 0.136 | 0.617 | 720 | 1.006 |
| ZI, Intercept | 0.268 | 0.495 | -0.697 | 1.282 | 949 | 1.006 |
| Count, Suicidal Thoughts, Between-Person | 0.259 | 0.115 | 0.039 | 0.486 | 841 | 1.017 |
| Count, Suicidal Thoughts, Within-Person | 0.032 | 0.019 | -0.005 | 0.070 | 906 | 1.015 |
| ZI, Suicidal Thoughts, Between-Person | -1.346 | 0.531 | -2.392 | -0.318 | 664 | 1.012 |
| ZI, Suicidal Thoughts, Within-Person | -0.049 | 0.071 | -0.185 | 0.085 | 1264 | 1.024 |
| Shape | 536.082 | 157.886 | 285.828 | 899.577 | 1511 | 1.024 |

*Note:* ZI = zero-inflated; SE = Standard Error, CI = Confidence Interval; ESS = Effective Sample Size; Shape is a dispersion parameter

Supplemental Table 9. Help-Seeking Dictionary Predicting Active Suicidal Ideation

| Parameter | Estimate | SE | 95% CI Lower | 95% CI Upper | Bulk ESS | Rhat |
| --- | --- | --- | --- | --- | --- | --- |
| Count, Random Intercept | 1.118 | 0.129 | 0.886 | 1.395 | 489 | 1.010 |
| ZI, Random Intercept | 4.525 | 0.582 | 3.562 | 5.828 | 503 | 1.017 |
| Count, Intercept | 0.053 | 0.149 | -0.245 | 0.344 | 994 | 1.009 |
| ZI, Intercept | -0.206 | 0.582 | -1.423 | 0.905 | 801 | 1.005 |
| Count, Help Seeking, Between-Person | 0.258 | 0.160 | -0.061 | 0.587 | 948 | 1.006 |
| Count, Help Seeking, Within-Person | -0.026 | 0.058 | -0.138 | 0.090 | 951 | 1.015 |
| ZI Help Seeking, Between-Person | 0.018 | 0.602 | -1.109 | 1.248 | 756 | 1.006 |
| ZI Help Seeking, Within-Person | 0.135 | 0.224 | -0.294 | 0.569 | 1102 | 1.008 |
| Shape | 450.954 | 159.151 | 231.888 | 841.193 | 1179 | 1.008 |

*Note:* ZI = zero-inflated; SE = Standard Error, CI = Confidence Interval; ESS = Effective Sample Size; Shape is a dispersion parameter

Supplemental Table 10. Hopelessness Dictionary Predicting Active Suicidal Ideation

| Parameter | Estimate | SE | 95% CI Lower | 95% CI Upper | Bulk ESS | Rhat |
| --- | --- | --- | --- | --- | --- | --- |
| Count, Random Intercept | 1.128 | 0.127 | 0.904 | 1.401 | 443 | 1.011 |
| ZI, Random Intercept | 4.453 | 0.569 | 3.514 | 5.677 | 550 | 1.012 |
| Count, Intercept | 0.046 | 0.161 | -0.270 | 0.352 | 725 | 1.004 |
| ZI, Intercept | -0.177 | 0.530 | -1.250 | 0.862 | 714 | 1.012 |
| Count, Hopeless, Between-Person | 0.072 | 0.134 | -0.190 | 0.342 | 745 | 1.009 |
| Count, Hopeless, Within-Person | -0.029 | 0.105 | -0.231 | 0.162 | 1019 | 1.006 |
| ZI, Hopeless, Between-Person | -0.506 | 0.602 | -1.745 | 0.627 | 604 | 1.009 |
| ZI, Hopeless, Within-Person | -0.025 | 0.409 | -0.865 | 0.764 | 1828 | 1.015 |
| Shape | 456.503 | 153.699 | 215.051 | 806.530 | 1418 | 1.009 |

*Note:* ZI = zero-inflated; SE = Standard Error, CI = Confidence Interval; ESS = Effective Sample Size; Shape is a dispersion parameter

Supplemental Table 11. Suicide Methods Dictionary Predicting Active Suicidal Ideation

| Parameter | Estimate | SE | 95% CI Lower | 95% CI Upper | Bulk ESS | Rhat |
| --- | --- | --- | --- | --- | --- | --- |
| Count, Random Intercept | 1.119 | 0.127 | 0.903 | 1.394 | 499 | 1.011 |
| ZI, Random Intercept | 4.514 | 0.609 | 3.499 | 5.853 | 592 | 1.005 |
| Count, Intercept | 0.053 | 0.153 | -0.260 | 0.333 | 814 | 1.000 |
| ZI, Intercept | -0.268 | 0.592 | -1.409 | 0.877 | 856 | 1.007 |
| Count, Suicide Methods, Between-Person | 0.106 | 0.164 | -0.222 | 0.439 | 824 | 1.004 |
| Count, Suicide Method,s Within-Person | -0.009 | 0.013 | -0.033 | 0.017 | 1134 | 1.001 |
| ZI, Suicide Methods, Between-Person | 0.415 | 0.624 | -0.781 | 1.799 | 738 | 1.008 |
| ZI, Suicide Methods, Within-Person | 0.104 | 0.049 | 0.010 | 0.199 | 952 | 1.010 |
| Shape | 455.003 | 167.870 | 214.326 | 848.576 | 1314 | 1.019 |

*Note:* ZI = zero-inflated; SE = Standard Error, CI = Confidence Interval; ESS = Effective Sample Size; Shape is a dispersion parameter

Supplemental Table 12. General Risk Dictionary Predicting Active Suicidal Ideation

| Parameter | Estimate | SE | 95% CI Lower | 95% CI Upper | Bulk ESS | Rhat |
| --- | --- | --- | --- | --- | --- | --- |
| Count, Random Intercept | 1.140 | 0.127 | 0.917 | 1.418 | 579 | 1.009 |
| ZI, Random Intercept | 4.193 | 0.568 | 3.235 | 5.426 | 611 | 1.013 |
| Count, Intercept | 0.035 | 0.153 | -0.268 | 0.339 | 792 | 1.004 |
| ZI, Intercept | -0.209 | 0.505 | -1.263 | 0.745 | 767 | 1.010 |
| Count, General Risk, Between-Person | 0.056 | 0.145 | -0.240 | 0.347 | 716 | 1.002 |
| Count, General Risk, Within-Person | 0.033 | 0.016 | 0.002 | 0.065 | 1030 | 1.025 |
| ZI, General Risk, Between-Person | -1.676 | 0.561 | -2.866 | -0.652 | 482 | 1.010 |
| ZI, General Risk, Within-Person | 0.079 | 0.060 | -0.040 | 0.190 | 860 | 1.013 |
| Shape | 443.618 | 141.501 | 224.422 | 790.211 | 1328 | 0.999 |

*Note:* ZI = zero-inflated; SE = Standard Error, CI = Confidence Interval; ESS = Effective Sample Size; Shape is a dispersion parameter

Supplemental Table 13. Sleep Dictionary Predicting Active Suicidal Ideation

| Parameter | Estimate | SE | 95% CI Lower | 95% CI Upper | Bulk ESS | Rhat |
| --- | --- | --- | --- | --- | --- | --- |
| Count, Random Intercept | 1.145 | 0.133 | 0.908 | 1.424 | 544 | 1.012 |
| ZI, Random Intercept | 4.454 | 0.599 | 3.483 | 5.823 | 370 | 1.018 |
| Count, Intercept | 0.036 | 0.161 | -0.272 | 0.354 | 829 | 0.998 |
| ZI, Intercept | -0.223 | 0.569 | -1.280 | 0.931 | 720 | 1.015 |
| Count, Sleep, Between-Person | 0.022 | 0.137 | -0.237 | 0.278 | 908 | 1.003 |
| Count, Sleep, Within-Person | 0.076 | 0.110 | -0.146 | 0.289 | 1043 | 1.013 |
| ZI, Sleep, Between-Person | -0.168 | 0.544 | -1.224 | 0.867 | 615 | 1.011 |
| ZI, Sleep, Within-Person | 0.188 | 0.414 | -0.661 | 0.978 | 2200 | 1.005 |
| Shape | 443.649 | 139.699 | 227.945 | 772.323 | 1177 | 1.009 |

*Note:* ZI = zero-inflated; SE = Standard Error, CI = Confidence Interval; ESS = Effective Sample Size; Shape is a dispersion parameter

Supplemental Table 14. Alcohol and Illicit Substances Dictionary Predicting Active Suicidal Ideation

| Parameter | Estimate | SE | 95% CI Lower | 95% CI Upper | Bulk ESS | Rhat |
| --- | --- | --- | --- | --- | --- | --- |
| Count, Random Intercept | 1.130 | 0.130 | 0.907 | 1.435 | 468 | 1.002 |
| ZI, Random Intercept | 4.363 | 0.581 | 3.366 | 5.614 | 490 | 1.011 |
| Count, Intercept | 0.040 | 0.153 | -0.275 | 0.315 | 765 | 1.013 |
| ZI, Intercept | -0.198 | 0.486 | -1.180 | 0.780 | 725 | 1.004 |
| Count, Alcohol & Illicit Substances, Between-Person | 0.099 | 0.161 | -0.217 | 0.430 | 787 | 1.007 |
| Count, Alcohol & Illicit Substances, Within-Person | -0.008 | 0.027 | -0.059 | 0.048 | 1088 | 1.009 |
| ZI, Alcohol & Illicit Substances, Between-Person | -1.033 | 0.615 | -2.362 | 0.141 | 703 | 1.008 |
| ZI, Alcohol & Illicit Substances, Within-Person | 0.013 | 0.107 | -0.206 | 0.227 | 1098 | 1.013 |
| Shape | 450.353 | 159.018 | 214.688 | 836.314 | 1489 | 1.004 |

*Note:* ZI = zero-inflated; SE = Standard Error, CI = Confidence Interval; ESS = Effective Sample Size; Shape is a dispersion parameter

Supplemental Table 15. Suicidal Thoughts Dictionary Predicting Active Suicidal Ideation

| Parameter | Estimate | SE | 95% CI Lower | 95% CI Upper | Bulk ESS | Rhat |
| --- | --- | --- | --- | --- | --- | --- |
| Count, Random Intercept | 1.104 | 0.130 | 0.880 | 1.388 | 506 | 1.011 |
| ZI, Random Intercept | 4.112 | 0.556 | 3.185 | 5.265 | 545 | 1.003 |
| Count, Intercept | 0.013 | 0.155 | -0.309 | 0.301 | 821 | 1.009 |
| ZI, Intercept | -0.090 | 0.504 | -1.103 | 0.841 | 533 | 1.020 |
| Count, Suicidal Thoughts, Between-Person | 0.237 | 0.142 | -0.035 | 0.516 | 797 | 1.013 |
| Count, Suicidal Thoughts, Within-Person | 0.018 | 0.021 | -0.023 | 0.059 | 1033 | 1.003 |
| ZI, Suicidal Thoughts, Between-Person | -1.527 | 0.527 | -2.556 | -0.495 | 485 | 1.013 |
| ZI, Suicidal Thoughts, Within-Person | -0.113 | 0.087 | -0.282 | 0.052 | 959 | 1.015 |
| Shape | 448.532 | 160.911 | 208.977 | 828.463 | 1753 | 1.013 |

*Note:* ZI = zero-inflated; SE = Standard Error, CI = Confidence Interval; ESS = Effective Sample Size; Shape is a dispersion parameter

Supplemental Table 16. Help-Seeking Dictionary Predicting Suicide Planning

| Parameter | Estimate | SE | 95% CI Lower | 95% CI Upper | Bulk ESS | Rhat |
| --- | --- | --- | --- | --- | --- | --- |
| Count, Random Intercept | 1.554 | 0.214 | 1.195 | 2.067 | 518 | 1.007 |
| ZI, Random Intercept | 3.527 | 0.552 | 2.655 | 4.781 | 381 | 1.033 |
| Count, Intercept | -0.600 | 0.251 | -1.097 | -0.148 | 677 | 1.000 |
| ZI, Intercept | 1.938 | 0.457 | 1.090 | 2.869 | 771 | 1.004 |
| Count, Help Seeking, Between-Person | 0.125 | 0.320 | -0.518 | 0.763 | 639 | 1.013 |
| Count, Help Seeking, Within-Person | 0.096 | 0.107 | -0.116 | 0.306 | 916 | 1.000 |
| ZI, Help Seeking, Between-Person | 0.539 | 0.550 | -0.479 | 1.703 | 638 | 1.010 |
| ZI, Help Seeking, Within-Person | 0.450 | 0.273 | -0.128 | 0.973 | 954 | 1.015 |
| Shape | 21.570 | 5.156 | 14.215 | 35.053 | 1013 | 1.001 |

*Note:* ZI = zero-inflated; SE = Standard Error, CI = Confidence Interval; ESS = Effective Sample Size; Shape is a dispersion parameter

Supplemental Table 17. Hopelessness Dictionary Predicting Suicide Planning

| Parameter | Estimate | SE | 95% CI Lower | 95% CI Upper | Bulk ESS | Rhat |
| --- | --- | --- | --- | --- | --- | --- |
| Count, Random Intercept | 1.532 | 0.215 | 1.175 | 2.008 | 513 | 1.013 |
| ZI, Random Intercept | 3.520 | 0.543 | 2.641 | 4.754 | 411 | 1.023 |
| Count, Intercept | -0.598 | 0.265 | -1.135 | -0.117 | 693 | 1.010 |
| ZI, Intercept | 1.890 | 0.483 | 0.913 | 2.856 | 627 | 1.008 |
| Count, Hopeless, Between-Person | -0.127 | 0.198 | -0.491 | 0.290 | 870 | 1.004 |
| Count, Hopeless, Within-Person | 0.073 | 0.180 | -0.285 | 0.419 | 1191 | 1.012 |
| ZI, Hopeless, Between-Person | -0.322 | 0.486 | -1.408 | 0.528 | 805 | 1.004 |
| ZI, Hopeless, Within-Person | -0.320 | 0.529 | -1.375 | 0.680 | 3000 | 1.010 |
| Shape | 21.960 | 5.848 | 14.037 | 36.225 | 1005 | 1.015 |

*Note:* ZI = zero-inflated; SE = Standard Error, CI = Confidence Interval; ESS = Effective Sample Size; Shape is a dispersion parameter

Supplemental Table 18. Suicide Methods Dictionary Predicting Suicide Planning

| Parameter | Estimate | SE | 95% CI Lower | 95% CI Upper | Bulk ESS | Rhat |
| --- | --- | --- | --- | --- | --- | --- |
| Count, Random Intercept | 1.583 | 0.239 | 1.202 | 2.146 | 332 | 1.012 |
| ZI, Random Intercept | 3.449 | 0.502 | 2.579 | 4.494 | 572 | 1.001 |
| Count, Intercept | -0.653 | 0.277 | -1.250 | -0.164 | 566 | 1.007 |
| ZI, Intercept | 1.884 | 0.479 | 0.972 | 2.803 | 796 | 1.003 |
| Count, Suicide Methods, Between-Person | 0.052 | 0.224 | -0.396 | 0.522 | 789 | 1.011 |
| Count, Suicide Methods, Within-Person | -0.001 | 0.025 | -0.052 | 0.046 | 1045 | 1.004 |
| ZI, Suicide Methods, Between-Person | -0.241 | 0.446 | -1.121 | 0.600 | 767 | 1.004 |
| ZI, Suicide Methods, Within-Person | 0.005 | 0.067 | -0.124 | 0.135 | 1166 | 1.004 |
| Shape | 21.615 | 5.206 | 14.044 | 33.614 | 1357 | 1.013 |

*Note:* ZI = zero-inflated; SE = Standard Error, CI = Confidence Interval; ESS = Effective Sample Size; Shape is a dispersion parameter

Supplemental Table 19. General Risk Dictionary Predicting Suicide Planning

| Parameter | Estimate | SE | 95% CI Lower | 95% CI Upper | Bulk ESS | Rhat |
| --- | --- | --- | --- | --- | --- | --- |
| Count, Random Intercept | 1.535 | 0.214 | 1.174 | 2.016 | 470 | 1.012 |
| ZI, Random Intercept | 3.482 | 0.523 | 2.589 | 4.737 | 622 | 1.005 |
| Count, Intercept | -0.601 | 0.251 | -1.142 | -0.162 | 589 | 1.012 |
| ZI, Intercept | 1.952 | 0.467 | 1.083 | 2.889 | 860 | 1.007 |
| Count, General Risk, Between-Person | -0.138 | 0.240 | -0.616 | 0.332 | 853 | 1.013 |
| Count, General Risk, Within-Person | 0.008 | 0.030 | -0.051 | 0.066 | 1120 | 1.000 |
| ZI, General Risk, Between-Person | -0.473 | 0.491 | -1.422 | 0.504 | 735 | 1.006 |
| ZI, General Risk, Within-Person | -0.268 | 0.074 | -0.415 | -0.121 | 1010 | 1.000 |
| Shape | 21.913 | 5.411 | 14.445 | 35.889 | 786 | 1.009 |

*Note:* ZI = zero-inflated; SE = Standard Error, CI = Confidence Interval; ESS = Effective Sample Size; Shape is a dispersion parameter

Supplemental Table 20. Sleep Dictionary Predicting Suicide Planning

| Parameter | Estimate | SE | 95% CI Lower | 95% CI Upper | Bulk ESS | Rhat |
| --- | --- | --- | --- | --- | --- | --- |
| Count, Random Intercept | 1.540 | 0.209 | 1.190 | 1.983 | 421 | 1.014 |
| ZI, Random Intercept | 3.439 | 0.510 | 2.564 | 4.578 | 370 | 1.025 |
| Count, Intercept | -0.616 | 0.261 | -1.154 | -0.125 | 480 | 1.021 |
| ZI, Intercept | 1.879 | 0.466 | 0.975 | 2.812 | 697 | 1.008 |
| Count, Sleep, Between-Person | -0.043 | 0.195 | -0.406 | 0.329 | 740 | 1.011 |
| Count, Sleep, Within-Person | 0.407 | 0.206 | 0.014 | 0.799 | 1160 | 1.011 |
| ZI, Sleep, Between-Person | -0.218 | 0.444 | -1.054 | 0.616 | 697 | 1.015 |
| ZI, Sleep, Within-Person | -0.089 | 0.498 | -1.055 | 0.900 | 3000 | 1.008 |
| Shape | 22.102 | 6.324 | 14.371 | 37.876 | 1089 | 1.010 |

*Note:* ZI = zero-inflated; SE = Standard Error, CI = Confidence Interval; ESS = Effective Sample Size; Shape is a dispersion parameter

Supplemental Table 21. Alcohol and Illicit Substances Dictionary Predicting Suicide Planning

| Parameter | Estimate | SE | 95% CI Lower | 95% CI Upper | Bulk ESS | Rhat |
| --- | --- | --- | --- | --- | --- | --- |
| Count, Random Intercept | 1.555 | 0.224 | 1.189 | 2.078 | 319 | 1.025 |
| ZI, Random Intercept | 3.448 | 0.531 | 2.591 | 4.650 | 470 | 1.019 |
| Count, Intercept | -0.617 | 0.269 | -1.204 | -0.134 | 485 | 1.020 |
| ZI, Intercept | 1.911 | 0.458 | 1.037 | 2.810 | 639 | 1.003 |
| Count, Alcohol & Illicit Substance, Between-Person | -0.003 | 0.234 | -0.469 | 0.468 | 775 | 1.008 |
| Count, Alcohol & Illicit Substance, Within-Person | -0.046 | 0.054 | -0.152 | 0.062 | 981 | 1.016 |
| ZI, Alcohol & Illicit Substance, Between-Person | -0.655 | 0.499 | -1.656 | 0.295 | 722 | 1.000 |
| ZI, Alcohol & Illicit Substance, Within-Person | -0.104 | 0.129 | -0.361 | 0.155 | 936 | 1.018 |
| Shape | 21.679 | 5.400 | 14.506 | 34.694 | 848 | 1.017 |

*Note:* ZI = zero-inflated; SE = Standard Error, CI = Confidence Interval; ESS = Effective Sample Size; Shape is a dispersion parameter

Supplemental Table 22. Suicidal Thoughts Dictionary Predicting Suicide Planning

| Parameter | Estimate | SE | 95% CI Lower | 95% CI Upper | Bulk ESS | Rhat |
| --- | --- | --- | --- | --- | --- | --- |
| Count, Random Intercept | 1.557 | 0.220 | 1.197 | 2.006 | 503 | 1.005 |
| ZI, Random Intercept | 3.353 | 0.530 | 2.496 | 4.564 | 367 | 1.021 |
| Count, Intercept | -0.676 | 0.270 | -1.228 | -0.171 | 600 | 1.014 |
| ZI, Intercept | 1.984 | 0.460 | 1.099 | 2.876 | 694 | 1.006 |
| Count, Suicidal Thoughts, Between-Person | 0.231 | 0.227 | -0.196 | 0.690 | 895 | 1.005 |
| Count, Suicidal Thoughts, Within-Person | -0.050 | 0.041 | -0.134 | 0.028 | 968 | 1.009 |
| ZI, Suicidal Thoughts, Between-Person | -0.675 | 0.440 | -1.554 | 0.218 | 663 | 1.010 |
| ZI, Suicidal Thoughts, Within-Person | -0.318 | 0.110 | -0.525 | -0.101 | 1075 | 1.020 |
| Shape | 21.727 | 5.174 | 14.222 | 34.387 | 794 | 1.018 |

*Note:* ZI = zero-inflated; SE = Standard Error, CI = Confidence Interval; ESS = Effective Sample Size; Shape is a dispersion parameter
